# Ileal neobladder adenocarcinoma occurring twenty-five years post ileocystoplasty: a case report

**DOI:** 10.11604/pamj.2023.44.149.38031

**Published:** 2023-03-28

**Authors:** Mayank Agrawal, Gaurav Malvi, Nirmit Agrawal, Supradeep Narayanaswamy, Rajvi Goradia, Bhushan Patil, Sujata Patwardhan

**Affiliations:** 1 Department of Urology, Seth Gordhandas Sunderdas (GS) Medical College and King Edward Memorial (KEM) Hospital, Mumbai, India

**Keywords:** Augmentation cystoplasty, adenocarcinoma, case report

## Abstract

De-tubularised ileum is one of the most common segments used for augmentation cystoplasty. It is associated with complications such as metabolic disturbances, recurrent urinary tract infections, and stone formation. However, adenocarcinoma arising in an augmented bladder is a rare occurrence. We report a 37-year-old female, case of ileocystoplasty 25 years ago due to a thimble bladder (genitourinary tuberculosis) who presented with hematuria for one month. Cystoscopy showed bladder mass in the transposed ileal segments. The patient underwent transurethral resection of the bladder lesion, and the histopathology was suggestive of adenocarcinoma of the ileum. Subsequently, she underwent anterior pelvic exenteration and post-operative recovery was uneventful. The 6-month follow-up showed that the patient was asymptomatic without recurrence. In conclusion, even though adenocarcinoma in the ileal neobladder is rare, life-long with close follow-up with routine cytologic, radiologic, and cystoscopic evaluation for early cancer detection and treatment at an early stage is crucial.

## Introduction

In tubercular small-capacity bladders, augmentation cystoplasty is usually done when nonsurgical management has failed [1]. Various types of gastroenteric segments have been described in the literature. De-tubularised ileum is one of the most common segments used for augmentation cystoplasty [2]. It is associated with complications such as metabolic disturbances, recurrent urinary tract infections, mucus production, and stone formation [2,3]. However, adenocarcinoma arising in an augmented bladder is a relatively rare occurrence [4,5]. We report a case of adenocarcinoma arising in an augmented bladder that occurred 25 years after ileocystoplasty.

## Patient and observation

**Patient information:** a 37-year-old lady presented with complaints of painless gross hematuria and dysuria for one month. In past, she had undergone augmentation ileocystoplasty at 12 years of age for a thimble bladder and left simple nephrectomy for pyonephrosis due to genitourinary tuberculosis. One year back she underwent a cystoscopy at an outside center for similar complaints that showed a tumor lesion measuring 1 x 1 cm and located at the bladder dome. The tumor was completely ablated endoscopically. There was no histopathology or urine cytology report available from that event. The patient did not have any associated chronic medical illness, or history of any substance abuse, or addiction. There was no family history of similar complaints.

**Clinical findings:** the patient´s vital signs and abdominal exam were normal.

**Figure 1:**
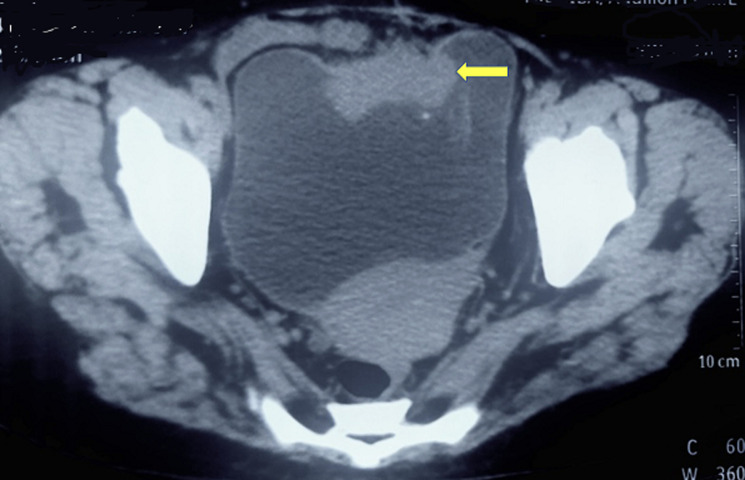
non-contrast computed tomography of kidney, ureter, and bladder region showing irregular, lobulated, enhancing soft tissue lesion along the anterior vesical wall measuring 2.1 x 4.7 x 4.8 cm with mild peri vesical fat stranding (arrow)

**Diagnostic assessment:** her blood investigations revealed hemoglobin of 12.4 g/dl and serum creatinine of 2.5 mg/dl. Liver function tests and serum electrolytes were in the normal range. Non-contrast computed tomography of the kidney, ureter, and bladder region (NCCT-KUB) revealed mild hydronephrosis and complete mild hydroureter of the right side. The urinary bladder was distended and showed a lobulated contour. There was an irregular, lobulated, enhancing soft tissue lesion along the anterior vesical wall in the transposed ileal segment measuring 2.1 x 4.7 x 4.8 cm with mild peri vesical fat stranding. There was no evidence of any enlarged lymph nodes on NCCT-KUB (Figure 1). Urine cytology was suspicious for malignant cells.

**Figure 2:**
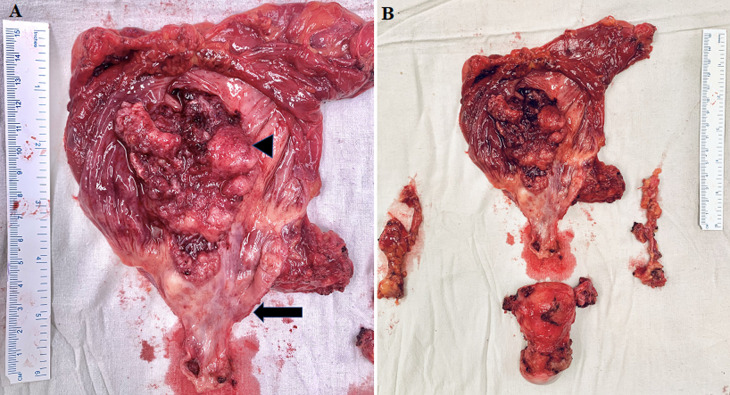
A) cut open specimen of augmented bladder and urethra showing ulcero-proliferative growth predominantly involving the bowel component of the augmented bladder (arrowhead); the native bladder did not show any changes on gross examination (arrow); B) radical cystectomy and hysterectomy specimen along with the lymph nodal packets

**Therapeutic intervention:** she underwent transurethral resection of bladder tumour (TURBT) under general anesthesia. On cystoscopy, there was a solid proliferative growth along the anterior vesical wall in the transposed ileal segment measuring 2 x 5 x 5 cm with evidence of neovascularization. The right ureteric orifice was not visible. The biopsy report was suggestive of adenocarcinoma of the ileum. The patient subsequently underwent anterior pelvic exenteration (open radical cystectomy and hysterectomy), and right-end ureterostomy (Figure 2). A decision for ureterostomy was taken considering her serum creatinine of 2.5 mg/dl and dilated thick-walled right ureter. The cut margin of the ureter was negative on the frozen section.

**Follow-up and outcomes:** the post-operative course was uneventful. The patient was discharged on post-operative day seven. The final histopathology report was suggestive of moderately differentiated adenocarcinoma, purely intestinal type. There was no urothelial differentiation. Adjacent mucosa showed intestinal metaplasia. Tumour infiltrated the deep muscle layer. All 16 lymph nodes examined were free of tumors. The right ureteric margin was also free of tumour (final stage - pT3N0M0). Flourine-18 fluorodeoxyglucose positron emission tomography/computed tomography (18F-FDG PET/CT) at 3 months postoperatively showed no evidence of abnormal uptake or suspicious metabolic active disease. At 6 months follow-up, the patient was asymptomatic with a serum creatinine of 2.2 mg/dl. A medical oncologist´s opinion was taken. The patient did not receive any adjuvant treatment in view of the absence of lymph nodal involvement and the absence of any metabolic active disease on FDG PET/CT.

**Patient perspective:** during her hospitalization and after treatment, the patient was satisfied with the care she received.

**Informed consent:** written informed consent was obtained from the patient for participation in our study.

## Discussion

Various types of gastro-enteric segments are used for augmentation of the small-capacity bladder. The ileum segment is the most common bowel segment used. The probability of developing a malignant tumor post-augmentation cystoplasty ranges from 0 to 5.5% [2]. Some authors have contemplated if the type of tissue used for the augmentation of the bladder has any relation with the risk of malignancy. Ries *et al*. suggested that there is a 7-8-fold increased risk of malignancy following an ileal or colonic augmentation and 14-15-fold following gastric augmentation [6]. However, Biardeau *et al*. found no significant difference in the incidence of cancer between the three different types of bowel segments used [2].

The carcinogenesis in the augmented bladder can be attributed to various mechanisms. Chronic inflammation because of bacteriuria is one factor. It results in the release of fibroblast growth factors, prostaglandin, eicosanoids, cytokines, and free oxygen radicals which can lead to carcinogenesis [2]. Secondly, the conversion of urinary nitrates to nitrites and finally to N-nitrosamines by the bacteria can induce deoxyribonucleic acid (DNA) mutagenesis [7,8]. Also, the cell-to-cell interactions because of anastomosis of two different kinds of epithelium (gastric/enteric with urothelium) can result in metaplasia and subsequent carcinogenesis [2]. Other associated factors like genetic predisposition, nicotine use, and immunosuppression also play important roles in carcinogenesis [8]. The common presenting symptoms in such cases are gross hematuria, lower urinary tract symptoms, and flank pain. Urine cytology, cystoscopic evaluation with tissue biopsy, and computed tomography scan help in reaching the diagnosis [2].

Although adenocarcinoma is the most common histology found in augmented bladders, varied histopathology has been reported, like urothelial carcinoma, squamous cell carcinoma, sarcomas, and small cell neuroendocrine tumors [2,9]. The latent period from the time of original surgery till the development of cancer ranged from 4 to 32 years. Heo *et al*. reported 2 cases of adenocarcinoma that developed in augmented bladders 23 and 30 years after ileocystoplasty, and both tumors were associated with contracted bladder due to tuberculosis [4]. Similarly, in our case, the histopathology was adenocarcinoma purely intestinal type. The reported data on the sub-type of adenocarcinoma found in augmented bladders is sparse. The malignancy can arise in either the native bladder or augmented segment or entero-urinary anastomotic line [7]. Biardeau *et al*. reported that in the majority (50%) of the cases, a tumor arises at the entero-urinary anastomosis [2]. Intestinal type is suggestive of adenocarcinoma arising from the epithelium of the bowel component of the augmented bladder and not the metaplasia of the urothelium of the bladder component to adenocarcinoma. Gross inspection of the specimen in our case also showed predominant involvement of the bowel component of the augmented bladder. The native bladder did not show any changes. A multimodal treatment approach is used for the management in the form of radical cystectomy, chemotherapy, and radiotherapy. It depends on the histology type and stage of the disease.

There are no recommended guidelines for surveillance [7,10]. Although the reported latent period between surgery and carcinogenesis is around 19-22 years, in our case it occurred after 25 years which emphasizes the need for lifelong surveillance [7,8]. Biardeau *et al*. pointed out that at present annual cystoscopy started 10 years after the surgery is the only reliable tool for surveillance [2]. So, it is important that it is done at a reliable center. In some cases, patients may go to any center of choice, and they may not be given standard treatment. In our case, the patient underwent cystoscopy and endo-ablation of a bladder tumor 1 year back at an outside center. The patient should be counseled that the check cystoscopy should be done by the primary operating team or at a tertiary care center where standard management protocols for such malignancies are established.

## Conclusion

Even though adenocarcinoma in the augmented ileal neobladder is rare, life-long with close follow-up with routine cytologic, radiologic, and cystoscopic evaluation for early cancer detection and treatment at an early stage is crucial. Patients' education and regular follow-up at specialized centers where standard management protocols for such malignancies are established are highly recommended.
